# 

**Published:** 1848-02

**Authors:** 


### CONTENTS

OF NO. I.

### ORIGINAL COMMUNICATIONS.

### ESSAYS AND CASES.

ART. I.—Notes on Medical Matters and Medical Men in Paris. By DAVID W. YANDELL, M.D., of Louisville, Ky..........93

ART. II.—A Case of Rupture of the Uterus with the escape of the Fœtus into the Abdomen. By JAMES M. LARKINS, M.D., of Charlotte, Tennessee..........110

### REVIEWS.

ART. III.—New Elements of Operative Surgery. By ALF. A. L. M. VELPEAU, Professor of Surgical Clinique of the Faculty of Medicine of Paris, &c., &c. Carefully revised, entirely remodelled, and augmented with a Treatise on Minor Surgery. Illustrated by over 200 Engravings, incorporated with the text. Accompanied with an Atlas in quarto of twenty-two plates, representing the principal operative processes, surgical instruments, &c. First American, from the last Paris edition. Translated by P. S. TOWNSEND, late Physician to the Seamen's Retreat, Staten Island, New York. Augmented by the addition of several hunded pages of entirely new matter, comprising all the latest improvements and discoveries in Surgery, in America and Europe, up to the present time. Under the supervision of, and with notes and observations by, VALÉNTINE MOTT, M.D., Professor of the Operations of Surgery with Surgical and Pathological Anatomy, in the University of New York, &c., &c. In three volumes. Vol. I. pp. 914: 1845. Vol. II. pp. 1092: 1846. Vol. III. pp. 1162: 1847. New York: Samuel S. & William Wood..........113

Art. IV.—Account of a New Anæsthetic Agent, as a Substitute for Sulphuric Ether in Surgery and Midwifery. By J. Y. Simpson, M.D., F.R.S.E., Professor Med. Univ. Edinburgh, &c. .......... 150

SELECTIONS FROM AMERICAN AND FOREIGN JOURNALS.

On the Convulsive Affections of Infants and Children. By Marshall Hall, M.D., F.R.S., Foreign Associate of thv Royal Academy of Medicine of Paris, etc., etc. .......... 153

ORIGINAL INTELLIGENCE.

Medical Department of the University of Louisville .......... 177
New Medical School in Louisville .......... 178
Visit from Dr. Cartwright.—Medical Statistics.—Epidemic Cholera. 182
Death of Mr. Liston .......... 184
Death of Dieffenbach .......... 184

